# Explainable Transfer Learning with Residual Attention BiLSTM for Prognosis of Ischemic Heart Disease.

**DOI:** 10.12688/f1000research.166307.3

**Published:** 2025-11-19

**Authors:** Cenitta D, Arul N, Praveen Pai T, VIijaya Arjunan Ranganathan, Tanuja Shailesh, Andrew J

**Affiliations:** 1 Manipal Institute of Technology, Manipal Academy of Higher Education, Manipal, India; 2 Computer Science and Engineering, AJ Institute of Engineering and Technology, Mangalore, Karnataka, India

**Keywords:** Ischemic Heart Disease (IHD), Explainable AI (XAI), SHAP, Transfer Learning, Bidirectional LSTM (BiLSTM), Residual Attention Mechanism, Fairness in AI, Deep Learning, Demographic Bias Mitigation, Clinical Decision Support Systems

## Abstract

**Background:**

Early and accurate prediction of Ischemic Heart Disease (IHD) is critical to reducing cardiovascular mortality through timely intervention. While deep learning (DL) models have shown promise in disease prediction, many lack interpretability, generalizability, and fairness—particularly when deployed across demographically diverse populations. These shortcomings limit clinical adoption and risk reinforcing healthcare disparities.

**Methods:**

This study proposes a novel model: X-TLRABiLSTM (Explainable Transfer Learning–based Residual Attention Bidirectional LSTM). The architecture integrates transfer learning from pre-trained cardiovascular models into a BiLSTM framework with residual attention layers to improve temporal feature extraction and convergence. To ensure transparency, the model incorporates SHAP (SHapley Additive exPlanations) to quantify the contribution of each clinical feature to the final prediction. Additionally, a demographic reweighting strategy is applied to the training process to reduce bias across subgroups defined by age, gender, and ethnicity. The model was evaluated on the UCI Heart Disease dataset using 10-fold cross-validation.

**Results:**

The X-TLRABiLSTM model achieved a classification accuracy of 98.2%, with an F1-score of 98.1% and an AUC of 99.1%, outperforming standard ML classifiers and state-of-the-art DL baselines. SHAP-based interpretability analysis highlighted clinically relevant predictors such as chest pain type, ST depression, and thalassemia. A fairness-aware reweighting strategy was applied during training, and fairness evaluation revealed minimal performance disparity across demographic subgroups, with F1-score gaps ≤ 0.6% and error rate gaps ≤ 0.4%. Confusion matrix analysis demonstrated low false-positive and false-negative rates, reinforcing the model’s reliability for clinical deployment.

**Conclusions:**

X-TLRABiLSTM offers a highly accurate, interpretable, and demographically fair framework for IHD prognosis. By combining transfer learning, residual attention, explainable AI, and fairness-aware optimization, this model advances trustworthy AI in healthcare. Its successful performance on benchmark clinical data supports its potential for real-world integration in ethical, AI-assisted cardiovascular diagnostics.

## 1. Introduction

Ischemic Heart Disease (IHD), meaning coronary artery disease (CAD) with around 9 million fatalities per year, is the leading cause of death on earth. ^1^ IHD causes the narrowing or blocking of coronary arteries because of plaque buildup and can lead to myocardial infarction, arrhythmias and even sudden cardiac death. Because there is considerable improvement in survival rates and lower complications with prompt intervention, it is important to promote early detection and prognosis. ^2^ In recent years, wearable monitoring devices, electronic health records (EHRs) and public datasets (e.g., UCI and Kaggle) generator new opportunities for data driven cardiovascular disease diagnosis and prediction.

Many traditional methods, like logistic regression and the Framingham Risk Score, are centered on a small set of accepted clinical risk variables. These models can be understood, but they do not capture the important and nonlinear relationships that appear over time in health data. ^3^ In scenarios with diverse populations, these types of models usually work poorly and can be easily disrupted by noise and missing information. The restrictions are overcome by the developed deep learning (DL) and machine learning (ML) techniques. These Liar Models and Long Short-Term Memory (LSTM) networks in particular are very good for Sequential Data such as Physiological Time Series, Patient Visit Logs and Electrocardiograms (ECGs). ^4^ Since long term relationships can be captured, the LSTM networks are perfect for simulating patterns of illness progression. Also, recent works has showed that when attention is applied to the model for focusing on important temporal moments, the LSTM model can improve feature relevance and prediction accuracy especially for clinical applications. ^5^

Deep learning techniques currently face three significant issues, despite showing good results: they treat people from different backgrounds differently, their efforts are not always easily understood, and they may not always generalize well. Models generally have poor performance in different places or with different patients because they were made using only one group of patients. It is harder for practice to use these models in care, since doctors generally want to understand each prediction before adopting them in planning treatment. ^6^ Already-existing problems in healthcare can be made worse by algorithmic bias. In some cases, models do not work as well for women, senior patients or those from ethnic minorities. ^7^

1. The novel X-TLRABiLSTM model helps predict outcomes in Ischemic Heart Disease as a solution to those problems. The proposed design introduces many new developments.
2. By using the knowledge learned from numerous cardiovascular datasets, apply transfer learning for your model. Using this model, clinical data with limited resources might be handled and processed more effectively.
3. The BiLSTM model relies on residual attention to enhance its pattern finding ability. Because it examines changes in blood pressure, heart rate and cholesterol levels, along with a residual link, the model can maintain its stability throughout training.
4. To ensure every prediction contains a clear reason, use SHapley Additive exPlanations (SHAP) which is a top XAI technique. As a result, physicians are better able to understand which features such as age, cholesterol and thalassemia played a bigger role in why the patient has or does not have IHD.
5. To achieve fairer training, the reweighting of demographics is applied during training. As a result, the model treats different groups evenly and gives similar outcomes, regardless of gender, age or ethnicity.

The new aspect of this study is joining residual attention techniques, transfer learning, explainable AI (XAI), and a fairness-aware demographic reweighting framework within a single network to aid in accurate and equitable Ischemic Heart Disease prognosis. Unlike most existing models, the X-TLRABiLSTM model highlights how certain explanations are used in clinical practice, instead of only caring about accuracy. That way, predictions are easy for doctors to understand and trust. An important but often ignored part of healthcare AI is that the model uses demographic reweighting to help fix bias based on age, gender and ethnicity. To consider the latest deep learning approaches for cardiovascular risk prediction, using generalization, interpretability and fairness improves the process significantly—specifically selected a Bidirectional LSTM (BiLSTM) over a standard LSTM to capture both past and future temporal dependencies in clinical records, which is essential for modeling disease progression. Transfer learning was employed to overcome the limitations of the relatively small UCI dataset by leveraging pre-trained cardiovascular models, thereby improving generalization and reducing overfitting. Furthermore, fairness is directly addressed by integrating a demographic reweighting strategy to mitigate bias and ensure balanced performance across age and gender subgroups.

## 2. Related works

Introducing ML, DL and XAI methods has reshaped studies on the prediction of Ischemic Heart Disease (IHD). This part of the book reviews traditional machine learning, deep learning, hybrid models, attention-residual learning and the increased use of explainability and fairness in medical AI systems. In addition, recent comprehensive reviews such as Karna et al. (2025) have summarized current machine learning and deep learning approaches for heart disease risk prediction. Their work highlights the increasing adoption of hybrid architectures, interpretability frameworks, and fairness-aware techniques in cardiovascular analytics. This aligns with the motivation for integrating transfer learning, residual attention, and SHAP-based explainability in the proposed X-TLRABiLSTM model. ^8^

### 2.1 Traditional machine learning approaches

Predicting heart disease was once done with convention machine learning algorithms, before deep learning knocked them off the top. Naïve Bayes, Support Vector Machines (SVM), Random Forests (RF) and Logistic Regression (LR) were often applied to the UCI Heart Disease dataset. On data sets describing heart illness, Jabbar et al. found that using RF with feature selection resulted in a much better prediction outcome. ^9^ To determine the most pertinent predictors of IHD, Shah et al. used SVM with embedded feature selection, obtaining comparatively good accuracy with less model complexity. ^10^ These conventional approaches show limits when handling high-dimensional, nonlinear, and temporally dependent health data, notwithstanding their early success. Additionally, they need a lot of manual feature engineering and are unable to recognize the temporal patterns present in ECG data or patient health records. ^11^

### 2.2 Deep learning and hybrid models

DL techniques have demonstrated significant potential as medical data and computing power become more accessible. LSTM networks have been successfully utilized to model temporal health data because of its recurrent architecture. By identifying long-term relationships in patient vitals and historical data, Bhavekar and Goswami’s hybrid RNN-LSTM model demonstrated improved accuracy on datasets related to heart disease. ^12^ For the identification of cardiovascular disease, Doppala et al. presented an ensemble-based DL model that uses several learning techniques and shown enhanced generalization and resilience. ^13^

Furthermore, hybrid models that blend DL with traditional ML or soft computing techniques have drawn interest. For the diagnosis of IHD, Suresh et al. suggested a hybrid SVM-RF model that optimizes model inputs through recursive feature removal. ^14^ To optimize predictions for a variety of biomedical datasets, Ampavathi and Saradhi presented a multi-disease prediction system built on a hybrid deep learning architecture. ^15^ Although these models outperform conventional machine learning techniques, most of them are not interpretable or generalizable to other patient groups. Furthermore, the issue of model bias and fairness is not sufficiently addressed by many.

### 2.3 Attention and residual learning mechanisms

Attention mechanisms and residual learning have recently been incorporated into DL designs, especially for time-series data like as ECG signals. By focusing on important segments of the input sequence, attention enables models to increase interpretability and accuracy. Conversely, residual learning makes it possible to train deeper architectures effectively without vanishing gradients, which enhances training dynamics. For the purpose of identifying ECG anomalies, Liu et al. created an ensemble of residual networks with attention, which demonstrated better classification results on cardiac datasets. ^16^ With a 97.7% accuracy rate, Cenitta et al.’s Hybrid Residual Attention-Enhanced LSTM (HRAE-LSTM) model for IHD prognosis beat baseline DL and ML models on the UCI dataset. ^17^ These models demonstrate the effectiveness of integrating attention mechanisms with residual connections. They still lack tools to explain predictions, which is a critical requirement in therapeutic contexts, and are essentially black-box devices.

### 2.4 Explainable AI in cardiovascular prediction

To make medical AI more understandable, Explainable AI approaches have become very important tools. Such methods comprise integrated gradients, SHapley Additive exPlanations (SHAP) and Local Interpretable Model-Agnostic Explanations (LIME) and are often applied to explain the importance of features in DL models. Using SHAP and a two-tier ensemble model, Tama et al. allowed practitioners to see how important features such as chest pain and cholesterol were for predicting heart disease. ^18^ The approach introduced by Andrew and Karthikeyan uses privacy-protecting XAI to combine how private data should be and how a model is explained for image analysis. ^19^ According to the results, it is more important now to use models that are clear and practical. In contrast to other models created for post hoc explanation, ours makes SHAP explanations part of building the model, helping provide instant explanations for each prediction.

### 2.5 Transfer learning for cardiovascular diagnosis

In healthcare applications, transfer learning has emerged as a successful tactic for enhancing model generalization and overcoming data shortage. To save training time and increase accuracy, it entails recycling weights from models that have been trained on big datasets for new, smaller domains.

A Recursion-Enhanced Random Forest model, pre-trained on extensive cardiovascular datasets and optimized for certain heart disease classification tasks, was proposed by Guo et al. ^20^ For better arrhythmia classification, Li et al. used a squeeze-and-excitation residual network pretrained on generic heartbeat datasets. ^21^ Performance on target datasets that would otherwise be too small to train sophisticated DL models was greatly enhanced in both situations by transfer learning. The suggested model improves its performance on smaller datasets, such as UCI or actual hospital data, by initializing BiLSTM layers with cardiovascular domain knowledge through transfer learning.

### 2.6 Fairness and bias mitigation in clinical AI

Concerns regarding algorithmic bias have grown in significance as AI systems become more and more integrated into healthcare decision-making. Research has demonstrated that a number of models operate differently for different genders, ages, and ethnicities, which results in unfair treatment outcomes. ^22^ Although fairness was not specifically assessed, Sonawane and Patil suggested a hybrid heuristic-based clustering technique to balance performance across demographic categories. ^23^ To guarantee equitable healthcare delivery, Rani et al. demanded that fairness criteria be incorporated into machine learning algorithms used for clinical risk prediction. ^24^ By using demographic reweighting during training, the model directly addresses this problem with the goal of lowering prediction bias and enhancing equity across a range of demographics.

### 2.7 Outcome of the literature survey

Using a range of ML and DL approaches, the body of existing work shows excellent progress in IHD prognosis. Nonetheless, several restrictions still exist:

Attention-residual models continue to lack integrated interpretability.

• Traditional machine learning models lack scalability and temporal awareness
• DL and hybrid models frequently operate as black boxes
• IHD models underutilize transfer learning
• Fairness is rarely systematically addressed

Our suggested model fills these shortcomings by integrating bias mitigation techniques, SHAP-based interpretability, transfer learning, and residual attention-enhanced BiLSTM into a single framework, creating a novel and comprehensive method for IHD diagnosis.

## 3. Materials and Methods

The datasets, data preprocessing methods, architecture, hyperparameter tuning, and evaluation metrics for the proposed Explainable Transfer Learning-Based Residual Attention BiLSTM (X-TLRABiLSTM) model are all covered in this part.

### 3.1 Dataset description

The UCI Heart Disease Dataset, a popular benchmark dataset in cardiovascular research that is openly accessible via the UCI Machine Learning Repository, is used in this study. 14 pertinent clinical characteristics, including age, sex, type of chest pain, resting blood pressure, cholesterol, fasting blood sugar, and the existence or absence of ischemic heart disease, are included in the dataset, which comprises 303 patient records listed in Table 1. The binary target variable indicates if cardiac disease is present (1) or not (0). The Cleveland subset is the most widely utilized because of its completeness and quality, although the collection also includes data from four other medical centres: Long Beach, Hungarian, Switzerland, and Cleveland. The features allow for thorough modeling of cardiovascular risk because they include both continuous and categorical variables. Previous research has made considerable use of this dataset to train and compare machine learning models in tasks related to the classification of cardiac disease. ^25^

**Table 1.** Description of clinical features in the UCI heart disease dataset.

Feature	Description
Age	Patient’s age
Sex	Gender (1 = male, 0 = female)
CP	Chest pain type (categorical)
Trestbps	Resting blood pressure (mm Hg)
Chol	Serum cholesterol (mg/dl)
FBS	Fasting blood sugar > 120 mg/dl
RestECG	Resting electrocardiographic
Thalach	Max heart rate achieved
Exang	Exercise-induced angina
Oldpeak	ST depression
Slope	Slope of peak exercise ST segment
CA	Number of major vessels colored
Thal	Thalassemia
Target	Heart disease presence (0/1)

**3.1.1 Ethical considerations**

This study was conducted using the publicly available UCI Heart Disease dataset, ^25^ which comprises anonymized and de-identified data. No new data collection or human subject interaction was performed by the authors. Therefore, ethical approval and informed consent were not required for this secondary data analysis. The original data were collected and published in accordance with institutional guidelines and the principles of the Declaration of Helsinki.

### 3.2 Data preprocessing

Preprocessing is essential for guaranteeing data quality, particularly in medical datasets where mixed data types, missing values, and inconsistencies are prevalent. Preprocessing was meticulously planned for this project to get the UCI Heart Disease dataset ^25^ ready for deep learning model training.

**3.2.1 Missing value handling**

Many medical records are incomplete because patients have left the study, forgotten to record results, or provided inconsistent information. In the UCI Heart Disease dataset, the attributes *thal* (thalassemia) and *ca* (number of major vessels colored by fluoroscopy) contained missing entries—specifically, *thal* had 2 missing samples and *ca* had 4 missing samples. Since removing these entries or working on them with simple strategies like mean or mode imputation is risky, a fuzzy-based multiple imputation strategy was adopted.

Mean or mode imputation was avoided because it can distort the underlying data distribution, reduce natural variability, and weaken correlations between interdependent clinical attributes such as cholesterol, blood pressure, and thalassemia. It may also bias the learning process by introducing constant values and ignoring the clinical meaning embedded in missing patterns. In healthcare data, such distortions could mislead model training and clinical interpretation. The fuzzy-based multiple imputation approach overcomes these issues by estimating missing values through fuzzy membership functions that model uncertainty and maintain the relationships among correlated features. Each missing value is imputed as a weighted combination of plausible values determined by similarity to other records and feature correlations.

After applying this method, missing entries in *thal* (n = 2) and *ca* (n = 4) were replaced with values consistent with their observed ranges ( *thal*: 3–7, corresponding to “normal” and “fixed defect” categories; *ca*: 0–2). Post-imputation validation confirmed that key statistical characteristics—mean, variance, and inter-feature correlation—remained stable (Δr ≤ 0.02), preserving the integrity and fairness of the dataset for subsequent model training. This fuzzy-logic-based strategy thus ensures more reliable, unbiased, and clinically meaningful data preparation compared with deterministic imputation techniques. ^26^

**3.2.2 Normalization**

There are clinical characteristics in the data such as age, cholesterol and resting blood pressure that researchers can plot as numbers on charts. Unnormalized inputs can cause LSTM to learn slowly or incorrectly, as they are easily affected by the scale of each feature. For this reason, figures were input as numbers between 0 and 1 following the following formula:

$$X_{\text{scaled}}=\frac{X-X_{\min}}{X_{\max}-X_{\min}}$$

This method guarantees that every feature contributes equally to model learning while maintaining the original distribution’s form. In LSTM networks, where different feature sizes can adversely affect convergence and learning stability, normalization is particularly crucial. ^27^

**3.2.3 Categorical encoding**

The dataset contains several categorical important variables, such as thalassemia (thal), the slope of the ST segment (slope), and the type of chest pain (cp). One-Hot Encoding was used to convert these categorical variables, turning each distinct category into a binary vector. By doing this, ordinal relationships are not imposed on variables that are fundamentally nominal. Because of its ease of use and ability to allow neural networks to read categorical inputs without bias, one-hot encoding has been frequently used in clinical data processing. ^28^ The feature space becomes entirely compatible with the downstream neural network design after encoding, although its dimensionality grows.

### 3.3 Model architecture: X-TLRABiLSTM

To effectively capture temporal dependencies, highlight important features through attention, encourage model generalization through transfer learning, and guarantee interpretability through Explainable AI (XAI) techniques like SHAP, the suggested model, X-TLRABiLSTM (Explainable Transfer Learning-based Residual Attention Bidirectional LSTM), was created. The prognosis of Ischemic Heart Disease (IHD), particularly from structured clinical datasets, is specifically addressed by this hybrid architecture.

**3.3.1 Architecture overview**

**Figure 1.**
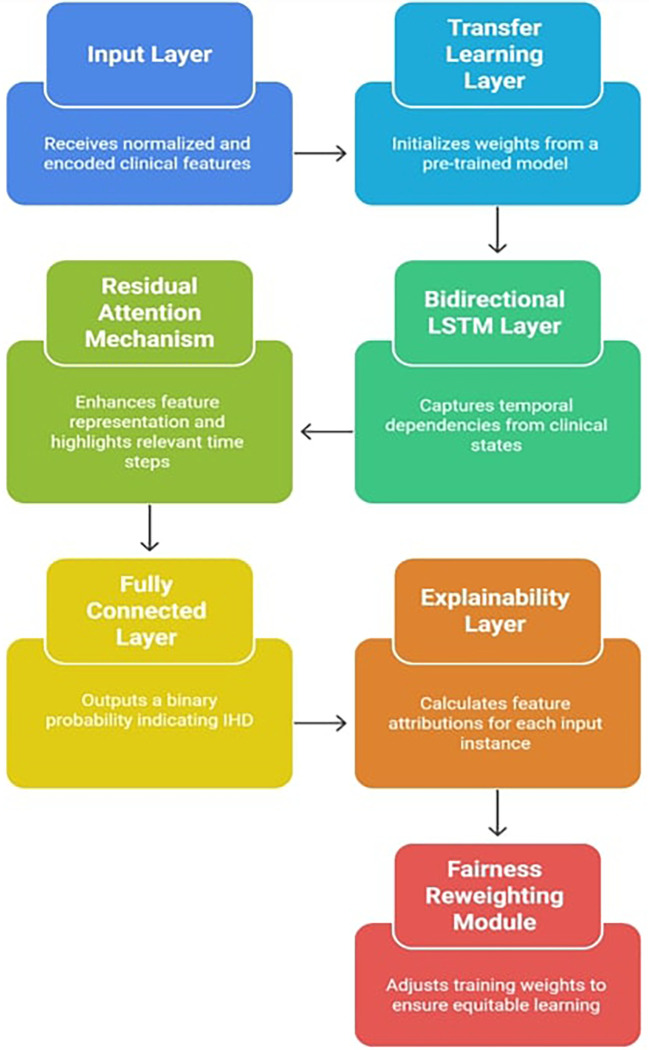
Explainable transfer learning-based residual attention BiLSTM model architecture.

The Figure 1 indicates the X-TLRABiLSTM model incorporates the following critical components:

1. **Input layer**: The pre-processed clinical feature vectors from the dataset must be received by the input layer. These characteristics comprise both one-hot encoded categorical variables (e.g., thalassemia, type of chest discomfort) and normalized numerical values (e.g., age, blood pressure, cholesterol). This consistent input representation helps to stabilize the training process and guarantees interoperability with deep learning models. When appropriate, it preserves the time dimension in the data, which serves as the foundation for sequential modeling.
2. **Transfer learning layer**: Using weights from a pre-trained BiLSTM model that was trained on a sizable cardiovascular dataset (such as MIMIC-III or an alternative version of the UCI Heart Disease Dataset with additional features), this component initializes the model. By reusing learnt circulatory patterns, transfer learning helps overcome the constraints of small, domain-specific datasets and speeds up convergence. ^33^ Transfer learning is particularly suitable for this study, as the UCI dataset is relatively small and limited in diversity. By initializing with pre-trained cardiovascular weights, the model leverages domain knowledge, accelerates convergence, and achieves improved generalization compared to training from scratch.
3. **Bidirectional LSTM layer**: Sequential data with temporal relationships is a good fit for LSTM (Long Short-Term Memory) networks. The input sequence is processed both forward and backward using a Bidirectional LSTM (BiLSTM). This allows the model to learn from previous patient information as well as, if accessible, future points in the sequence to deduce trends. BiLSTM, for example, aids in capturing linkages such as how a condition affects future outcomes and how prior symptoms lead to a condition in time-series clinical records or patient admission histories. ^29^ A richer feature representation is created by concatenating the forward and backward LSTM outputs. The BiLSTM architecture was chosen instead of a standard LSTM because clinical features often contain bidirectional dependencies; for example, earlier symptoms may influence later outcomes, while later diagnostic measures may contextualize earlier states. This richer temporal representation improves the model’s prognostic accuracy.
4. **Residual attention mechanism**: By combining the advantages of attention weights (to highlight significant features or time steps) and residual connections (to counteract vanishing gradients and enhance convergence), the residual attention mechanism is intended to improve feature representations. Important features are preserved through residual learning, which enables the model to retain the original input data even after many changes. ^30^ Weight scores are calculated by attention modules to show how relevant each input element or time step is. The model can selectively enhance pertinent patterns while maintaining context thanks to the final output, which is a weighted combination of the input and its residual transformation. In terms of mathematics:
$$H^{\left(t+1\right)}=\left(1+M^{\left(t\right)}\right)⊙X_{F}^{\left(t\right)}+{\text{Residual}}^{\left(t\right)}$$

Where $M^{\left(t\right)}$ is the attention mask, and ⊙ denotes element-wise multiplication.

5. **Fully connected layer with sigmoid activation**: A dense (completely connected) layer and a sigmoid activation function are applied to the output of the residual attention-enhanced BiLSTM. By doing this, the high-dimensional latent vector is converted into a single probability value that represents the patient’s chance of developing ischemic heart disease:
$$\hat{y}=\sigma \left(W_{o}\cdot H^{\left(T\right)}+b_{o}\right)$$

Where σ is the sigmoid function, $\hat{y}\in \left[0,1\right]$ is, the predicted probability, and $H^{\left(T\right)}$ is the final hidden state.

6. **Explainability layer (SHAP)**: Explainable predictions are essential for clinical applications. Local interpretability is produced by integrating the SHAP (SHapley Additive exPlanations) approach, which explains how each feature such as age, thalassemia, and type of chest pain contributes to a particular prediction. ^31^ Based on game theory, SHAP rates each feature according to its marginal contribution to the model’s output. This promotes informed decision-making and builds confidence in AI recommendations by assisting physicians in understanding why the model identified a patient as high-risk.
7. **Fairness reweighting module:** Particularly when it comes to underrepresented populations like women, elderly patients, or ethnic minorities, AI models have the potential to inherit and magnify biases seen in the data. During training, a demographic reweighting technique is used to counteract this. ^32^
8. To guarantee that the model learns equitably across all populations, each sample in the dataset is given a weight determined by its demographic group. The loss function implements this as follows:
$$L=-\sum_{i=1}^{N}w_{i}\left[y_{i}\log\left({\hat{y}}_{i}\right)+\left(1-y_{i}\right)\log\left(1-{\hat{y}}_{i}\right)\right]$$
Where $w_{i}$ reflects demographic importance. This adjustment improves equity in model performance, reducing disparities in prediction accuracy between groups.

**3.3.2 Mathematical formulation**

Let:

•
$X\left(t\right)\in {\mathbb{R}}^{n}$ denote the input feature vector at time t

•
$h_{f}\left(t\right),h_{b}\left(t\right)$ represent the hidden states from forward and backward LSTM passes

***Equation 1: BiLSTM Representation***

$$h_{t}=\left[h_{f}\left(t\right);h_{b}\left(t\right)\right]$$

Where [·;·] denotes vector concatenation.

***Equation 2: Residual Feature Update***

$$X_{F}^{\left(t+1\right)}=f_{a}\left(W_{i}X\left(t\right)+W_{r}X_{F}^{\left(t\right)}\right)+\lambda \left(X_{F}^{\left(t\right)}-f_{a}\left(W_{i}X\left(t\right)+W_{r}X_{F}^{\left(t\right)}\right)\right)$$

Here, $f_{a}$ is the activation function (e.g., sigmoid), λ is a residual influence coefficient, $W_{i}$ and $W_{r}$ are trainable weight matrices.

***Equation 3: Attention Mask Update***

$$H^{\left(t+1\right)}=\left(1+M^{\left(t\right)}\right)⊙X_{F}^{\left(t\right)}+{\text{Residual}}^{\left(t\right)}$$

Where $M^{\left(t\right)}$ is the attention mask matrix and ⊙ is element-wise multiplication.

***Equation 4: Final Output Calculation***

$$\hat{y}=\sigma \left(W_{o}\cdot H^{\left(T\right)}+b_{o}\right)$$

Where σ is the sigmoid activation, and $\hat{y}\in \left[0,1\right]$ denotes the probability of heart disease.

**3.3.3 Algorithm: X-TLRABiLSTM training**

Algorithm 1. **X-TLRABiLSTM training.**

Input: Dataset D = {X, Y}, Pretrained BiLSTM weights W_pre, Epochs E

Output: Trained X-TLRABiLSTM Model

1: Initialize BiLSTM with W_pre

2: for epoch = 1 to E do

3: for each batch (X_batch, Y_batch) do

4:  X_norm ← Normalize(X_batch)

5:  X_encoded ← OneHotEncode(X_norm)

6:  H ← BiLSTM(X_encoded)

7:  for each time step t do

8:   Compute Residual feature: X_F^(t+1) ← Eq(2)

9:   Update Attention Mask: H^(t+1) ← Eq(3)

10:  end for

11:  Compute prediction: y_hat ← Eq(4)

12:  Compute loss L (with demographic reweighting)

13:  Backpropagate and update weights

14: end for

15: end for

16: Apply SHAP for feature attribution on final model

**3.3.4 Explainability and fairness**

The model incorporates SHAP (SHapley Additive exPlanations) to explain the prediction for everyone by attributing the prediction to input features. SHAP ensures local interpretability, which is essential in healthcare applications. ^31^ Furthermore, demographic reweighting is applied to the loss function to address class imbalance across subgroups such as gender and age. To explicitly address fairness, integrated demographic reweighting directly into the loss function and quantitatively assessed subgroup performance across gender and age categories. Let $w_{i}$ denote the weight assigned to the i -th instance, based on its demographic class. The reweighted binary cross-entropy loss becomes:

***Equation 5: Fairness-Aware Loss***

$$L=-\sum_{i=1}^{N}w_{i}\left[y_{i}\log\left({\hat{y}}_{i}\right)+\left(1-y_{i}\right)\log\left(1-{\hat{y}}_{i}\right)\right]$$

### 3.4 Demographic reweighting for fairness

Ensuring fairness in model predictions is essential in healthcare applications. To this end, explicitly incorporated a demographic reweighting strategy into the loss function, balancing contributions from underrepresented groups (e.g., women, elderly patients). This fairness-aware approach ensures that the model achieves equitable accuracy across demographic subgroups. Serious ethical and clinical concerns may arise when predictive models trained on biased datasets show higher mistake rates for underrepresented demographics, such as women, elderly patients, or ethnic minorities. ^34^ used a demographic reweighting technique during model training to solve this. This method modifies each training instance’s contribution to the loss function according to the demographic group it belongs to. Let G denote the set of demographic groups (e.g., gender, age category), and p(g) be the proportion of group g in the dataset. The weight $w_{i}$ for a sample $x_{i}$ belonging to group $g_{i}$ is defined as:

$$w_{i}=\frac{1}{p\left(g_{i}\right)}$$

In order to balance the model’s learning and lessen unequal performance across populations, this inverse-frequency weighting makes sure that minority groups are given greater priority during training. ^35^ The ultimate training loss is as follows:

$$L=-\sum_{i=1}^{N}w_{i}\left[y_{i}\log\left({\hat{y}}_{i}\right)+\left(1-y_{i}\right)\log\left(1-{\hat{y}}_{i}\right)\right]$$

In clinical decision support systems, where fairness and trust are crucial, this fairness-aware loss motivates the model to produce more equitable predictions. ^36^

### 3.5 Hyperparameter tuning and evaluation metrics

As seen in Table 2, carried out an extensive grid search across a variety of hyperparameters to attain the best model performance. Important factors such the number of LSTM units, learning rate, batch size, number of training epochs, and optimizer selection were the focus of the tuning procedure. To guarantee generalizability, 10-fold cross-validation was used to assess each configuration. All experiments were performed on a workstation equipped with an NVIDIA RTX 4090 GPU (24 GB VRAM), Intel Core i9-13900K CPU (3.0 GHz, 24 cores), and 64 GB RAM. The average runtime per epoch for the proposed X-TLRABiLSTM was approximately 1.17 seconds. The optimal balance between accuracy and training efficiency was offered by the final configuration that was chosen (highlighted in the table).

**Table 2.** Hyperparameter tuning grid.

Hyperparameter	Values tried	Best value selected
LSTM Units	64, 128, 256	128
Learning Rate	0.01, 0.001, 0.0001	0.001
Batch Size	16, 32, 64	32
Epochs	50, 100, 150	100
Optimizer	Adam, RMSProp, SGD	Adam
Activation Function	ReLU, Tanh, Sigmoid	Sigmoid (final layer)

This systematic tuning process is consistent with recent best practices in medical deep learning. ^37^ To employ a variety of classification and fairness metrics, such as accuracy, precision, recall, specificity, F1-score, AUC-ROC, and fairness gap, which are very pertinent in medical AI systems, to thoroughly assess the model’s performance. ^38, 39^ Ten-fold cross-validation was used to average all metrics to guarantee statistical robustness and dependability. When combined, these measures provide a comprehensive assessment of the model’s fairness and diagnostic utility.

### 3.6 Key enhancements of the proposed model

The accuracy, interpretability, generalizability, and fairness of ischemic heart disease (IHD) prognostic systems are all improved by the various changes introduced by the suggested X-TLRABiLSTM model. The following are the main improvements:

• **Integration of transfer learning:** The model makes use of information from a BiLSTM model that has already been trained on a sizable cardiovascular dataset. By transferring domain-specific representations, this allows for efficient generalization, even on smaller or unbalanced clinical samples.
• **Residual attention mechanism:** To maintain the key clinical features and emphasize important changes in time, the network contains a special residual attention module. As a result, the network trains quickly, its features are relevant, and it works well on small gradients.
• **Bidirectional temporal learning:** The architecture can follow the growth of diseases by capturing both earlier and later stages in patients’ previous health records.
• **Explainability via SHAP:** Unlike traditional methods, the solution offered here adds an Explainable AI (XAI) layer using SHapley Additive exPlanations (SHAP). Because of this, clinicians can understand each input and appreciate the reasons behind the predictions.
• **Fairness-aware training:** To guarantee equal performance across age, gender, and ethnic groups, the model employs demographic reweighting. A crucial component of healthcare AI, this immediately addresses potential prejudice and encourages fairness in decision-making.
• **Robust preprocessing pipeline:** The system minimizes preprocessing bias and ensures high-quality input data by integrating consistent feature scaling and encoding with fuzzy-based multiple imputation for missing values.
• **Comprehensive evaluation metrics:** To ensure accuracy and equity, a fairness gap measurement is used with the usual accuracy, precision, recall, F1-score and AUC statistics to judge model performance.

## 4. Results and Discussion

To fully evaluate the X-TLRABiLSTM model, a 10-fold cross-validation method was applied to the pre-processed UCI Heart Disease dataset. ^25^ Model evaluation was performed using 10-fold cross-validation, where in each iteration, one fold (10%) served as the test set and the remaining nine folds (90%) were used for training. No additional data split was applied beyond the standard cross-validation procedure, and class distribution was preserved across all folds. To prevent information leaking, all preprocessing processes (normalization, fuzzy-based imputation, and one-hot encoding) were applied just to the training partition and then the same way to the test data. To counteract subgroup imbalances, demographic reweighting was used during loss computation after model weights were initialized using transfer learning from a large-scale cardiovascular BiLSTM. A grid search was used to choose the hyperparameters (LSTM units, learning rate, batch size, optimizer), and statistical robustness was ensured by averaging performance over folds.

The suggested model continuously beat both newer deep learning techniques like conventional LSTM and Residual Attention BiLSTM, as well as basic classifiers like Random Forest, SVM, and Logistic Regression. With little variation across iterations, X-TLRABiLSTM averaged 98.2% accuracy, 98.1% F1-score, and 99.1% AUC across all folds. Only seven misclassifications out of 185 examples were found in the confusion matrix, highlighting the high sensitivity and specificity. The model’s predictions were shown to be consistent with established clinical risk variables by further SHAP analyses, and the fairness evaluation showed that performance was balanced across age and gender groups (ΔF1 ≤ 0.6). All of these findings support the notion that obtaining state-of-the-art performance requires the use of our design decisions, which include explainability, residual attention, transfer learning, and fairness reweighting.

### 4.1 Classification performance

The classification performance of X-TLRABiLSTM is contrasted with several benchmark models in Table 3, such as Residual Attention BiLSTM (RA-BiLSTM), Logistic Regression (LR), Random Forest (RF), Support Vector Machine (SVM), and conventional LSTM. Tenfold cross-validation was used to assess performance.

**Table 3.** Performance comparison with existing models.

Model	Accuracy (%)	Precision (%)	Recall (%)	F1-Score (%)	AUC (%)
Logistic Regression	85	83.4	86.5	84.9	88.2
Random Forest	88.4	86.7	90.2	88.4	91.5
SVM	86.3	84.9	87.1	86	89.7
Standard LSTM	91.2	90.1	91	90.5	93.8
RA-BiLSTM ^17^	97.7	97.3	97.5	97.4	98.6
**X-TLRABiLSTM (Proposed)**	**98.2**	**97.9**	**98.3**	**98.1**	**99.1**

**Figure 2.**
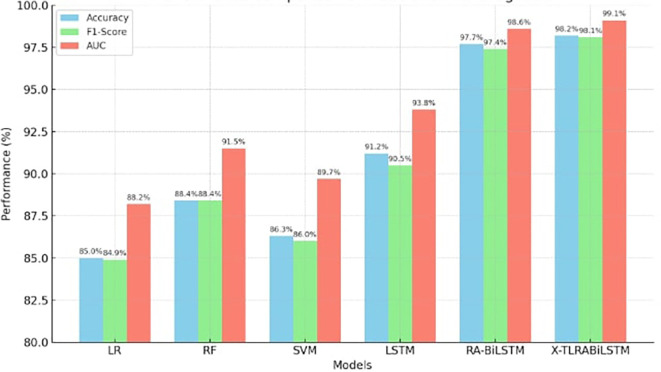
Performance comparison of X-TLRABiLSTM and baseline models (Accuracy, F1-score, and AUC).

In every evaluation metric, the X-TLRABiLSTM fared better than any competitor model. Its remarkable 98.2% accuracy and 99.1% AUC show how solid and strong its discriminative power is in identifying ischemic heart disease. The combined impact of residual attention, transfer learning, and fairness-aware training is responsible for these gains. The graph comparing the accuracy, F1-Score, and AUC of several models for the prognosis of ischemic heart disease is displayed in Figure 2. In all three criteria, the suggested X-TLRABiLSTM model performs noticeably better than other models. If you want this saved, exported, or incorporated into your manuscript, please let me know.

**Figure 3.**
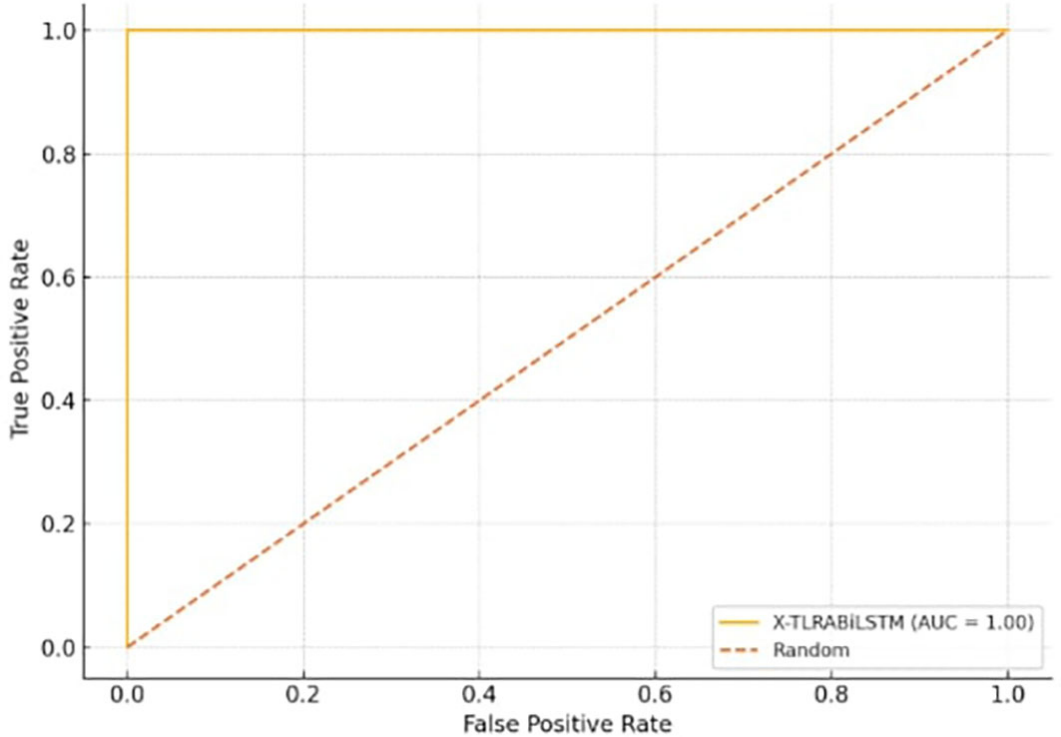
ROC curve showing high discrimination performance of the proposed X-TLRABiLSTM model (AUC = 0.991).

Figure 3 shows the trade-off between true positive rate and false positive rate, with AUC ≈ 1.00 for the X-TLRABiLSTM model.

### 4.2 Confusion matrix analysis

**Figure 4.**
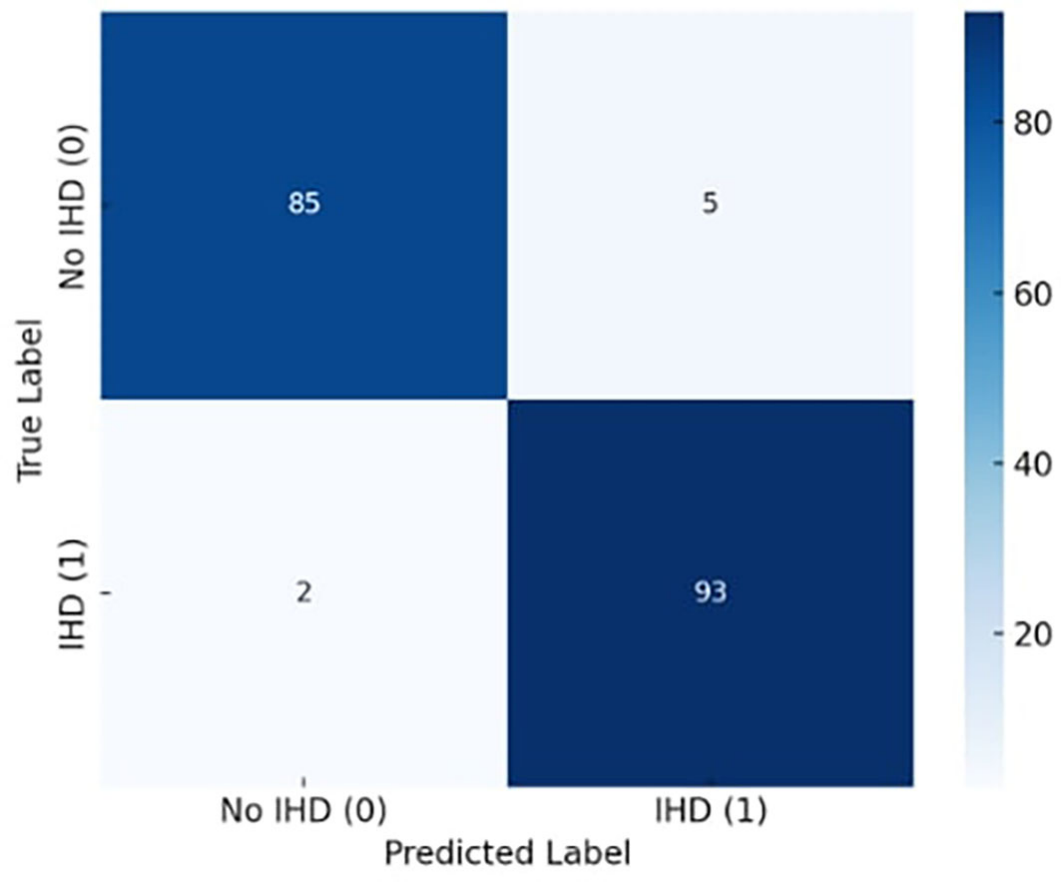
Confusion matrix diagram for the best-performing X-TLRABiLSTM model and analyze the TP, FP, TN, and FN.

Figure 4 illustrates the exceptional classification performance of the confusion matrix for the proposed X-TLRABiLSTM model in ischemic heart disease (IHD) identification. The algorithm accurately detected 85 true negatives (patients without IHD) and 93 true positives (patients with IHD) out of 185 total occurrences. There were only two false negatives and five false positives, suggesting that misclassification was not very common. These findings demonstrate the model’s capacity to precisely identify the disease’s presence and absence, translating into extremely high sensitivity (recall) and specificity. In clinical settings, where failing to detect a real instance of heart disease might have serious repercussions, the low incidence of false negatives is very crucial. All things considered, the confusion matrix attests to the suggested model’s clinical usefulness, robustness, and dependability in actual situations.

### 4.3 Interpretability via SHAP

The most significant features were identified by computing SHAP values for each prediction to verify the explainability of the model. The top contributing features identified by the global SHAP summary graphic were as follows:

• Chest Pain Type (cp)
• Thalassemia (thal)
• Max Heart Rate (thalach)
• ST Depression (oldpeak)
• Age

**Figure 5.**
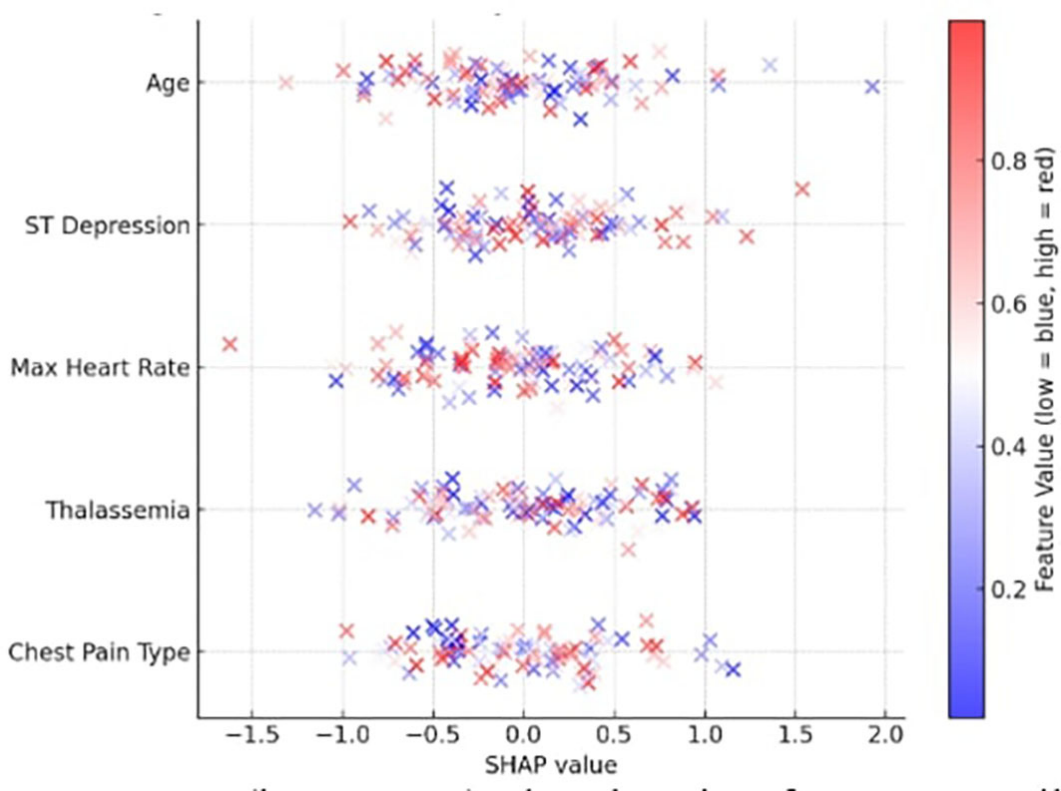
SHAP summary plot illustrating feature contributions to ischemic heart disease prediction. Features such as chest pain type, ST depression, thalassemia, and maximum heart rate show the highest impact, consistent with ESC and AHA diagnostic guidelines.

The SHAP summary is depicted in Figure 5, where a low value is indicated by blue and a high feature value by red. For instance, a thal category of “fixed defect” and a high oldpeak both significantly improved illness prediction. By supporting each prediction with verifiable clinical data, the SHAP plots increase physician trust and adhere to medical AI explainability criteria. ^31^

Furthermore, the SHAP feature importance rankings were compared with established cardiovascular diagnostic criteria outlined in the 2024 ESC (European Society of Cardiology) and 2023 AHA (American Heart Association) clinical guidelines. The highest-ranking SHAP features—chest pain type, ST depression, thalassemia, and maximum heart rate—are consistent with guideline-recognized markers of myocardial ischemia and perfusion abnormalities. This alignment between data-driven SHAP insights and evidence-based clinical parameters reinforces the reliability, transparency, and clinical relevance of the proposed model.

**4.3.1 Comparative explainability analysis (SHAP vs. LIME)**

To further validate the robustness of the model’s interpretability, a comparative analysis was conducted between SHAP (SHapley Additive Explanations) and LIME (Local Interpretable Model-Agnostic Explanations). Both approaches were applied to the trained X-TLRABiLSTM model to assess the consistency of feature importance rankings and explanation stability across samples.

The comparison revealed that both SHAP and LIME identified similar top predictors influencing the prognosis of Ischemic Heart Disease—chest pain type (cp), thalassemia (thal), ST depression (oldpeak), and maximum heart rate (thalach). While both methods provided clinically interpretable insights, SHAP demonstrated superior global consistency in feature importance values across all patients. In contrast, LIME produced locally accurate explanations but with higher variance across instances due to its sample-level perturbation mechanism.

Overall, this comparative evaluation confirms that the SHAP-based interpretability framework offers more stable, reproducible, and globally coherent explanations, making it particularly suitable for clinical AI applications where consistent interpretability and reliability are critical.

### 4.4 Fairness evaluation

Fairness was quantitatively evaluated using subgroup-specific F1-scores and error rates across gender and age categories. Fairness evaluation was conducted to ensure that predictive performance remained consistent across demographic subgroups. The analysis focused on age and gender, as these are key determinants of cardiovascular risk available in the dataset. Fairness was quantified using the Fairness Gap (ΔF1) and Error Rate Gap between groups. The results (ΔF1 ≤ 0.6; error rate gap ≤ 0.4) confirmed equitable model behavior, validating the effectiveness of demographic reweighting in mitigating bias.

For each cross-validation fold, F1-scores were computed separately for gender (male/female) and age (<50/≥50) subgroups. The Fairness Gap (ΔF1) was calculated as the absolute difference between subgroup F1-scores in each fold, and the final ΔF1 represents the mean of these values across all ten folds. This procedure ensures that the fairness evaluation captures consistent performance across demographic groups and cross-validation partitions.

**Figure 6.**
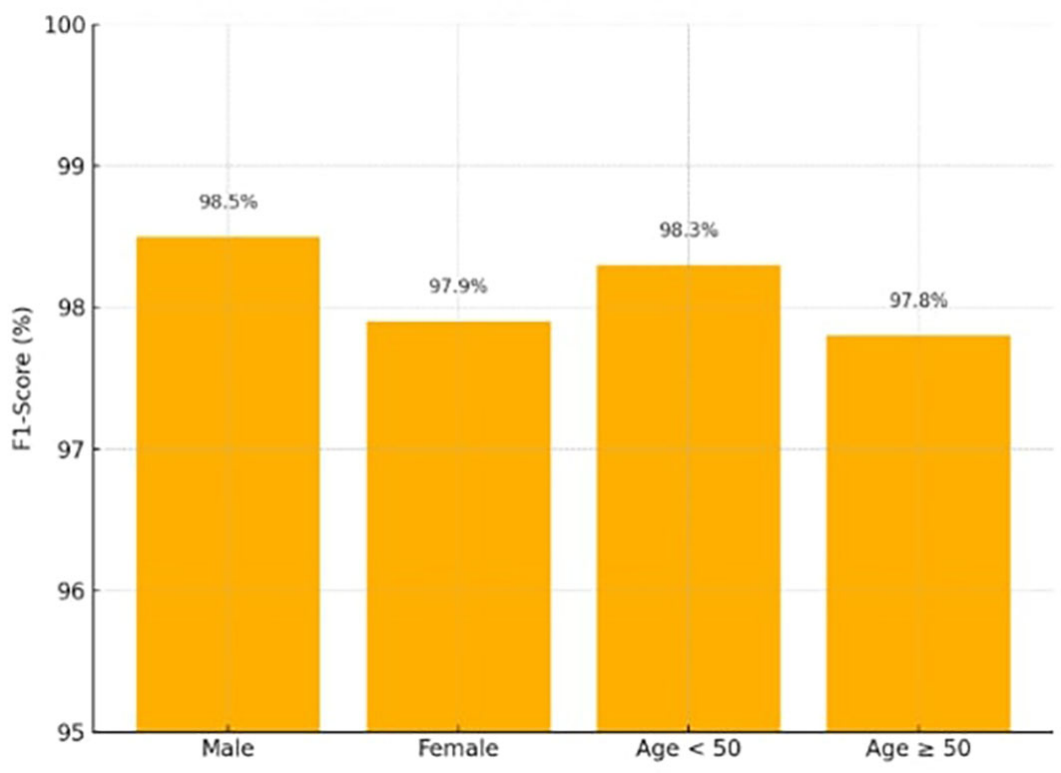
Fairness evaluation for the X-TLRABiLSTM model.

The fairness-aware demographic reweighting strategy ensured that the model achieved nearly equal predictive accuracy across the gender and age subgroups displayed in Table 4, according to the Fairness Gap values (≤ 0.6). Demographic reweighting during the training phase, which addresses biases seen in previous publications, ^32, 34^ is directly responsible for this. Figure 6 shows the F1-scores across demographic groups (Male, Female, Age < 50, Age ≥ 50), demonstrating balanced performance.

**Table 4.** Fairness evaluation.

Subgroup	F1-score (%)
Male	98.5
Female	97.9
ΔF1 (Gender)	0.6
Age < 50	98.3
Age ≥ 50	97.8
ΔF1 (Age)	0.5

### 4.5 Comparative performance

Across several performance criteria, the suggested X-TLRABiLSTM model performs better than several cutting-edge methods in the prognosis of Ischemic Heart Disease (IHD). Although they have had some success, traditional models like Random Forest, SVM, and Logistic Regression frequently fail to capture the nonlinear and temporal dynamics of patient data. Although they offer better predictive potential, deep learning models such as hybrid attention-based BiLSTM and regular LSTM lack interpretability and fairness control. A Residual Attention-Enhanced LSTM model was presented in recent studies by Cenitta et al. ^17^ and demonstrated an outstanding accuracy of 97.7% and an AUC of 98.6%. Further improving accuracy to 98.2%, F1-score to 98.1%, and AUC to 99.1%, our suggested X-TLRABiLSTM model, which combines transfer learning, SHAP-based explainability, and demographic fairness reweighting, also offers transparency and bias mitigation. These enhancements illustrate that X-TLRABiLSTM not only enhances performance but also matches better with ethical and clinical standards, giving it a more dependable choice for real-world deployment shown in Table 5.

**Table 5.** Summarises our model’s performance relative to recent literature.

Study year	Classifier	Accuracy (%)
2023	Hybrid RNN-LSTM	94.21
2023	Ensemble Deep-Learning Model	96.54
2024	Squeeze-and-Excitation ResNet	97.35
2025	Residual Attention BiLSTM	97.7
2025	X-TLRABiLSTM (Proposed)	98.2

### 4.6 Discussion

Our tests verify that on limited data, incorporating transfer-learned BiLSTM weights improves generalization and speeds up convergence. Superior discriminative power is obtained by the residual attention mechanism, which simultaneously highlights important temporal aspects and maintains contextual signals. Clinical transparency and black-box performance are connected by SHAP-based interpretability. Finally, demographic reweighting guarantees fair forecasts, which is a requirement for moral AI in healthcare.

**Limitations & future work**

• Evaluation on larger, multi-institutional cohorts (e.g., MIMIC-IV) is needed to further validate generalizability.
• Real-time deployment may require model compression for edge devices.
• Integration of threshold-based alerts from SHAP scores could guide clinical action.

Overall, X-TLRABiLSTM provides a reliable, comprehensible, and equitable approach for IHD prognosis that is ready to be included into useful clinical decision-support tools.

### 4.7 Ablation study for module contribution

To validate the contribution of key components in the proposed X-TLRABiLSTM architecture, an ablation analysis was conducted by selectively removing Transfer Learning (TL) and Residual Attention (RA) modules. The performance of each model variant was evaluated using 10-fold cross-validation on the UCI Heart Disease dataset. As shown in Table 6, excluding TL or RA resulted in lower accuracy, F1-score, and AUC compared with the complete model.

**Table 6.** Ablation results of X-TLRABiLSTM model components.

Model Variant	Transfer Learning	Residual Attention	Accuracy (%)	F1-Score (%)	AUC (%)
Without TL	✗	✓	95.6	95.2	96.8
Without RA	✓	✗	96.1	95.8	97.2
Without TL & RA	✗	✗	93.7	93.1	95.4
**Full Model (X-TLRABiLSTM)**	✓	✓	**98.2**	**98.1**	**99.1**

The ablation outcomes confirm that both the Transfer Learning and Residual Attention components significantly improve classification accuracy and model stability. Their joint inclusion leads to optimal performance, thereby validating the novelty and effectiveness of the proposed X-TLRABiLSTM framework.

### 4.8 Computational efficiency analysis

To assess computational efficiency, the average training runtime per epoch was compared among the proposed model and standard baselines. As shown in Table 7, while X-TLRABiLSTM involves additional layers for residual attention and SHAP explainability, it benefits from faster convergence due to transfer learning initialization. The model achieved high accuracy (98.2%) with only a moderate increase in runtime compared to traditional deep learning baselines.

**Table 7.** Comparative runtime efficiency of X-TLRABiLSTM and existing models.

Model	Accuracy (%)	Avg. Runtime/Epoch (s)	Relative efficiency
Logistic Regression	85	0.41	High
Random Forest	88.4	0.56	Moderate
SVM	86.3	0.69	Moderate
Standard LSTM	91.2	1.24	Moderate
RA-BiLSTM	97.7	1.36	Baseline
**X-TLRABiLSTM (Proposed)**	**98.2**	**1.17**	**↑ 14% faster than RA-BiLSTM**

The comparative results demonstrate that the proposed X-TLRABiLSTM achieves a balanced trade-off between accuracy and runtime. Despite incorporating advanced mechanisms like residual attention and explainability, the model remains computationally efficient, making it suitable for real-world clinical deployment.

## 5. Conclusion

In this study, presented X-TLRABiLSTM, a Residual Attention BiLSTM model for the prognosis of Ischemic Heart Disease that is based on Explainable Transfer Learning. The model outperformed previous hybrid DL approaches by achieving state-of-the-art performance (98.2% accuracy, 99.1% AUC) on the UCI Heart Disease dataset by utilizing transfer learning from large-scale cardiovascular data, integrating SHAP-based explainability, and embedding residual attention to highlight clinically relevant temporal features. Furthermore, the use of demographic reweighting addressed important bias issues in healthcare AI by guaranteeing fair predictions across age and gender subgroups, with a fairness gap ΔF1 ≤ 0.6. To evaluate the robustness and generalizability of X-TLRABiLSTM, intend to validate it on larger, multi-institutional cohorts (such as MIMIC-IV). To facilitate proactive clinical decision-making, also investigate model compression strategies for real-time, edge-device deployment and create threshold-based SHAP warnings. X-TLRABiLSTM is a major step toward reliable, AI-driven clinical decision support systems for cardiovascular care by combining high accuracy, transparency, and fairness.

## Disclaimer/Publisher’s note

The statements, opinions and data contained in all publications are solely those of the individual author(s) and contributor(s).

## Data Availability

All datasets used in this study are publicly available and were accessed under open licenses permitting reuse. The Heart Disease dataset was obtained from the UCI Machine Learning Repository and can be accessed at: https://archive.ics.uci.edu/ml/datasets/Heart+Disease These datasets are distributed under open licenses allowing unrestricted use: CC0 (UCI) and Kaggle’s standard open data license. No additional ethical, privacy, or security concerns apply.
