# Supplementary material for: Explainable Transfer Learning with Residual Attention BiLSTM for Prognosis of Ischemic Heart Disease
Source: F1000Res. 2025 Nov 19;14:651. Originally published 2025 Jul 4. [Version 3] doi: 10.12688/f1000research.166307.3 (PMC12596549; doi:10.12688/f1000research.166307.3)
Supplement: Peer review history

# Reviewer report: Reviewer response for version 3

**DOI:** 10.5256/f1000research.191512.r434284

**Published:** 2025-11-26

**Authors:** Ramesh Chandra Poonia

**Affiliations:** 1 Department of Computer Science, Christ University (Ringgold ID: 585354), Bangalore, Karnataka, India; 2 CHRIST, Delhi, India

The author has revised the manuscript as per the comments, and it is acceptable for indexing.

Is the work clearly and accurately presented and does it cite the current literature?

Yes

If applicable, is the statistical analysis and its interpretation appropriate?

Yes

Are all the source data underlying the results available to ensure full reproducibility?

Yes

Is the study design appropriate and is the work technically sound?

Yes

Are the conclusions drawn adequately supported by the results?

Yes

Are sufficient details of methods and analysis provided to allow replication by others?

Yes

Reviewer Expertise:

Cyber Physication Systems and Artifical Intelligence

I confirm that I have read this submission and believe that I have an appropriate level of expertise to confirm that it is of an acceptable scientific standard.

# Reviewer report: Reviewer response for version 2

**DOI:** 10.5256/f1000research.188562.r424480

**Published:** 2025-11-08

**Authors:** Karna Vishnu Vardhana Reddy

**Affiliations:** 1 Aditya University, Surampalem, Andhra Pradesh, USA

Please provide the number of samples having missing values from ‘thal’ and ‘ca’ attributes.

 Why the mean or mode imputation is risky? Please discuss in what way it is risky in detail.

 The study should compare SHAP with at least one other interpretability approach (for example:  LIME, Integrated Gradients) to show robustness.

 How is it overcome using fuzzy-based imputation strategy? Discuss about the results (the replaced missing values) obtained after applying fuzzy-logic method.

 What exactly the fairness evaluation in this work, discuss clearly. Why it was performed only on age, gender features?

 To justify novelty, the authors must show quantitative ablation results (example model variants without transfer learning or without attention) to confirm each module’s contribution.

 Provide a comparative runtime or computational efficiency analysis versus existing architectures.

 Figures 2, 3, 5 require better resolution, labeling, and clinical relevance discussion (example linking SHAP feature rankings to clinical guidelines such as ESC or AHA criteria).

 Explain how “fairness gap” (ΔF1) was computed across folds.

 Clarify hardware specifications used for training (GPU/CPU type, RAM, runtime).

 In standard 10-fold cross-validation, the entire dataset is automatically split into 10 folds. In each iteration, one fold is used for testing and the remaining nine folds (90%) are used for training. So, the 90%-10% split** *is inherent* in 10-fold CV - it doesn’t need to be separately stated. However, the wording *“the data was divided into training (90%) and testing (10%) sets in each fold”* **can mislead readers. Clarify whether 10-fold cross-validation itself was used as the sole evaluation strategy, or if a separate 90/10 split preceded the folds.

Is the work clearly and accurately presented and does it cite the current literature?

Yes

If applicable, is the statistical analysis and its interpretation appropriate?

I cannot comment. A qualified statistician is required.

Are all the source data underlying the results available to ensure full reproducibility?

No source data required

Is the study design appropriate and is the work technically sound?

Yes

Are the conclusions drawn adequately supported by the results?

Yes

Are sufficient details of methods and analysis provided to allow replication by others?

Yes

Reviewer Expertise:

Biomedical applications, machine learning, deep learning, Artificial Intelligence

I confirm that I have read this submission and believe that I have an appropriate level of expertise to confirm that it is of an acceptable scientific standard, however I have significant reservations, as outlined above.

## References

[1] : A Comprehensive Review on Heart Disease Risk Prediction using Machine Learning and Deep Learning Algorithms. *Archives of Computational Methods in Engineering* .2025;32(3) : 10.1007/s11831-024-10194-4 1763-1795 10.1007/s11831-024-10194-4

# Response

**Published:** 2025-11-14

**Authors:** VIJAYA ARJUNAN RANGANATHAN

**Affiliations:** School of Computer Engineering, Manipal Academy of Higher Education, Manipal, Karnataka, India

An Explainable Transfer Learning based Residual Attention BiLSTM Model for Fair and Accurate Prognosis of Ischemic Heart Disease

**Responses to peer review reports**

**Reviewer#3, Concern # 1:** Please provide the number of samples having missing values from ‘thal’ and ‘ca’ attributes.

**Author response:**  We thank the reviewer for this helpful observation. In the UCI Heart Disease (Cleveland) dataset used in our study, the attributes ‘thal’ (thalassemia) and ‘ca’ (number of major vessels colored by fluoroscopy) contain missing entries. Specifically,

- ‘thal’ has 2 missing samples, and
- ‘ca’ has 4 missing samples.

**Author action: **We have added the explicit counts of missing samples for the ‘thal’ and ‘ca’ attributes in Section 3.2.1 in the revised manuscript for clarity.



**Reviewer#3, Concern # 2:** Why the mean or mode imputation is risky? Please discuss in what way it is risky in detail.

**Author response:**  We appreciate the reviewer’s insightful question. Mean or mode imputation, while simple, can introduce several **statistical and clinical risks** in medical datasets such as the UCI Heart Disease dataset. Specifically: Distortion of Data Distribution, Loss of Correlation Structure, Bias in Predictive Modelling, and Clinical Interpretation Errors.

**Author action: **We have expanded the explanation in Section 3.2.1 to describe why mean/mode imputation is risky and how fuzzy-based multiple imputation mitigates these issues.



**Reviewer#3, Concern # 3:** The study should compare SHAP with at least one other interpretability approach (for example:  LIME, Integrated Gradients) to show robustness.

**Author response:** We thank the reviewer for this valuable suggestion. We agree that comparing SHAP with another interpretability approach can strengthen the robustness of our explainability assessment. Accordingly, we have performed an additional comparative analysis between SHAP and LIME  to demonstrate the stability and consistency of feature importance rankings.

**Author action: **We have added a new subsection titled “Comparative Explainability Analysis (SHAP vs. LIME)” under Section 4.3 Interpretability via SHAP, describing the comparison results and interpretation consistency.



**Reviewer#3, Concern # 4: **How is it overcome using fuzzy-based imputation strategy? Discuss about the results (the replaced missing values) obtained after applying fuzzy-logic method.

**Author response:**  We thank the reviewer for the valuable comment. The fuzzy-based multiple imputation strategy effectively overcomes the limitations of mean or mode imputation by preserving data variability and inter-feature correlations. Missing values in *thal* (n=2) and *ca* (n=4) were replaced with values consistent with their clinical ranges, maintaining the dataset’s statistical integrity (Δr ≤ 0.02).

**Author action: **We have elaborated Section 3.2.1 (Missing value handling) to include a brief explanation of how fuzzy-based multiple imputation operates and the imputed outcomes for *thal* and *ca*.



**Reviewer#3, Concern # 5: **What exactly the fairness evaluation in this work, discuss clearly. Why it was performed only on age, gender features?

**Author response:**  We appreciate the reviewer’s insightful query. Fairness evaluation in this study assesses whether the model performs equitably across demographic subgroups. It was conducted on age and gender because these are the only demographic features available in the UCI dataset and are clinically relevant to IHD risk. The fairness-aware reweighting approach minimized disparities (ΔF1 ≤ 0.6; error rate gap ≤ 0.4), confirming unbiased model behavior.

**Author action: **We have revised Section 4.4 to explicitly define fairness assessment, explain why it was applied to age and gender, and clarify how subgroup performance metrics were computed.



**Reviewer#3, Concern # 6: **To justify novelty, the authors must show quantitative ablation results (example model variants without transfer learning or without attention) to confirm each module’s contribution.

**Author response:**  We thank the reviewer for this valuable suggestion. A quantitative ablation study was added to demonstrate the contribution of each module. Results show that removing transfer learning or residual attention reduces performance (accuracy drops from 98.2% to 95.6% and 96.1%, respectively), confirming that both modules significantly enhance model generalization and validate the novelty of the proposed X-TLRABiLSTM architecture.

**Author action: **We have added a new subsection titled “4.7 Ablation Study for Module Contribution” under the Results and Discussion section.



**Reviewer#3, Concern # 7: **Provide a comparative runtime or computational efficiency analysis versus existing architectures.

**Author response:**  We appreciate the reviewer’s insightful suggestion. A comparative runtime analysis was added to evaluate the computational efficiency of the proposed model against baseline architectures. Results show that X-TLRABiLSTM achieves 14% faster convergence than RA-BiLSTM while maintaining the highest accuracy (98.2%), confirming its effectiveness and suitability for clinical deployment.

**Author action: **We have added a new subsection titled “4.8 Computational Efficiency Analysis” following the ablation study in the Results and Discussion section.



**Reviewer#3, Concern # 8: **Figures 2, 3, 5 require better resolution, labeling, and clinical relevance discussion (example linking SHAP feature rankings to clinical guidelines such as ESC or AHA criteria).

**Author response:**  We thank the reviewer for this valuable suggestion. Figures 2, 3, and 5 have been enhanced with improved resolution (300 dpi), clear labelling, and uniform formatting for better readability. Additionally, the SHAP-based interpretability section was expanded to link top-ranked features—such as chest pain type, ST depression, thalassemia, and maximum heart rate—with established ESC (2024) and AHA (2023) clinical guidelines, reinforcing the model’s clinical relevance.

**Author action: **Figures 2, 3, and 5 were replaced with high-resolution versions featuring consistent labelling and improved clarity. A new paragraph was added at the end of Section 4.3 (Interpretability via SHAP) to discuss the clinical significance of SHAP feature rankings in alignment with ESC and AHA ischemic heart disease diagnostic criteria.



**Reviewer#3, Concern # 9: **Explain how “fairness gap” (ΔF1) was computed across folds.

**Author response:**  We thank the reviewer for this important query. The Fairness Gap (ΔF1) was computed by calculating F1-scores for each demographic subgroup (male/female and age <50/≥50) in every cross-validation fold. The ΔF1 value represents the absolute difference between subgroup F1-scores, averaged across all ten folds to ensure consistency and robustness of fairness evaluation.

**Author action: **A detailed explanation of the ΔF1 computation method has been added in Section 4.4, clarifying that subgroup-specific F1-scores were compared per fold and averaged across folds to derive the final Fairness Gap value.



**Reviewer#3, Concern # 10: **Clarify hardware specifications used for training (GPU/CPU type, RAM, runtime).

**Author response:**  We appreciate the reviewer’s suggestion. Hardware details have been added for clarity and reproducibility. Model training was conducted on an NVIDIA RTX 4090 GPU, Intel Core i9-13900K CPU, and 64 GB RAM, with an average runtime of 1.17 seconds per epoch.

**Author action: **Hardware specifications were included in Section 3.5 and cross-referenced in Section 4.8.



**Reviewer#3, Concern # 11:** In standard 10-fold cross-validation, the entire dataset is automatically split into 10 folds. In each iteration, one fold is used for testing and the remaining nine folds (90%) are used for training. So, the 90%-10% split *is inherent* in 10-fold CV - it doesn’t need to be separately stated. However, the wording *“the data was divided into training (90%) and testing (10%) sets in each fold” *can mislead readers. Clarify whether 10-fold cross-validation itself was used as the sole evaluation strategy, or if a separate 90/10 split preceded the folds.

**Author response:**  We appreciate the reviewer’s clarification. Only 10-fold cross-validation was used as the evaluation strategy — no separate 90/10 split preceded it. The 90%-10% division refers solely to the internal folds within cross-validation.

**Author action: **The sentence in Section 4 was revised to clearly state that 10-fold cross-validation alone was employed, with no additional dataset split.



**Reviewer#3, Concern # 12:** **References**

 Karna V, Karna V, Janamala V, Devana V, et al.: A Comprehensive Review on Heart Disease Risk Prediction using Machine Learning and Deep Learning Algorithms. *Archives of Computational Methods in Engineering*. 2025; **32** (3): 1763-1795

**Author response:**  We thank the reviewer for pointing out this reference. The suggested citation by Karna et al. (2025) has been verified and incorporated into the revised manuscript to strengthen the related work and contextual background on recent advances in heart disease prediction using machine learning and deep learning.

**Author action: **The reference has been added in Section 2.



# Reviewer report: Reviewer response for version 1

**DOI:** 10.5256/f1000research.183275.r411313

**Published:** 2025-09-17

**Authors:** Ramesh Chandra Poonia

**Affiliations:** 1 Department of Computer Science, Christ University (Ringgold ID: 585354), Bangalore, Karnataka, India; 2 CHRIST, Delhi, India

Suggestions for Improvement:

1. The phrase “Fair and Accurate Prognosis” could be shortened to just “Accurate Prognosis” unless fairness is explicitly addressed with a defined framework or metrics.
2. Consider slightly restructuring for brevity, e.g.,
*“Explainable Transfer Learning with Residual Attention BiLSTM for Prognosis of Ischemic Heart Disease”*
  or
*“Explainable Residual Attention BiLSTM with Transfer Learning for Accurate Ischemic Heart Disease Prognosis”*
3. Ensure that the paper includes a strong justification for each component mentioned in the title (e.g., why BiLSTM over standard LSTM, why transfer learning is suitable for the dataset, and how fairness is ensured).

Is the work clearly and accurately presented and does it cite the current literature?

Yes

If applicable, is the statistical analysis and its interpretation appropriate?

Yes

Are all the source data underlying the results available to ensure full reproducibility?

Yes

Is the study design appropriate and is the work technically sound?

Yes

Are the conclusions drawn adequately supported by the results?

Yes

Are sufficient details of methods and analysis provided to allow replication by others?

Yes

Reviewer Expertise:

Computational Intelligence, Cyber Physical Systems, Machine Learning and Artificial Intelligence

I confirm that I have read this submission and believe that I have an appropriate level of expertise to confirm that it is of an acceptable scientific standard, however I have significant reservations, as outlined above.

# Response

**Published:** 2025-09-21

**Authors:** VIJAYA ARJUNAN RANGANATHAN

**Affiliations:** School of Computer Engineering, Manipal Academy of Higher Education, Manipal, Karnataka, India

**Reviewer#2, Concern # 1:** The phrase “Fair and Accurate Prognosis” could be shortened to just “Accurate Prognosis” unless fairness is explicitly addressed with a defined framework or metrics.

**Author response:**  We thank the reviewer for this observation. In our study, fairness is not only a conceptual point but is explicitly addressed through a demographic reweighting strategy and quantitative fairness evaluation. Specifically, we applied fairness-aware loss functions during training and reported subgroup-level performance (ΔF1 ≤ 0.6, error rate gap ≤ 0.4) across gender and age categories. These results demonstrate that the model achieves equitable performance across demographic groups, thereby justifying the inclusion of the term “Fair” in the title.

**Author action: **We have clarified this more explicitly in the abstract, introduction, and results sections to highlight the fairness framework and metrics applied in the study. For example, we now state in the abstract that fairness evaluation revealed minimal disparity across demographic subgroups, and we emphasize in the methods/results that fairness-aware optimization was implemented and quantitatively validated.



**Reviewer#2, Concern # 2:** Consider slightly restructuring for brevity, e.g.,

*“Explainable Transfer Learning with Residual Attention BiLSTM for Prognosis of Ischemic Heart Disease”*

 or

*“Explainable Residual Attention BiLSTM with Transfer Learning for Accurate Ischemic Heart Disease Prognosis”*

**Author response:**  We thank the reviewer for this helpful suggestion and agree that a more concise title improves readability.

**Author action: **The title has been revised to:

 “Explainable Transfer Learning with Residual Attention BiLSTM for Prognosis of Ischemic Heart Disease.”



**Reviewer#2, Concern # 3:** Ensure that the paper includes a strong justification for each component mentioned in the title (e.g., why BiLSTM over standard LSTM, why transfer learning is suitable for the dataset, and how fairness is ensured).

**Author response:**  We thank the reviewer for this constructive suggestion. We agree that the rationale for each component of the proposed framework should be clearly articulated. Our revised manuscript now emphasizes:

- Why BiLSTM over standard LSTM: BiLSTM captures both forward and backward temporal dependencies, which is critical in modelling clinical progression patterns where both prior and subsequent states provide diagnostic cues. This leads to richer feature representations compared to standard LSTM.
- Why transfer learning is suitable: The UCI Heart Disease dataset is relatively small and limited in diversity. Transfer learning from pre-trained cardiovascular models improves generalisation, accelerates convergence, and reduces overfitting on small datasets.
- How fairness is ensured: A demographic reweighting strategy was integrated into the loss function, ensuring balanced performance across subgroups (age and gender). We quantitatively validated fairness with subgroup-specific F1-scores and error rates (ΔF1 ≤ 0.6, error rate gap ≤ 0.4).

**Author action: **We revised the Introduction (Section 1) to include clearer motivation for BiLSTM, transfer learning, and fairness. Additionally, we expanded the Methods (Sections 3.3.1 and 3.4) to justify these components explicitly and highlighted the fairness evaluation more clearly in the Results (Section 4.4).



# Reviewer report: Reviewer response for version 1

**DOI:** 10.5256/f1000research.183275.r411315

**Published:** 2025-09-16

**Authors:** Krishna Kumar Joshi, Neelam Joshi

**Affiliations:** 1 Computer Engineering, Fr. Conceicao Rodrigues Institute of Technology, Navi Mumbai, Maharashtra, India; 2 Rajiv Gandhi Proudyogiki Vishwavidyalaya, MP, India

In this paper, the authors propose an **X-TLRABiLSTM-**based approach for predicting Ischemic Heart Disease (IHD) that strikes a balance between accuracy, interpretability, and fairness. Using the UCI Heart Disease dataset, the model combines three key elements: transfer learning from pre-trained BiLSTM models, a residual attention mechanism to capture essential temporal patterns, and SHAP-based explanations to highlight critical risk factors. To mitigate demographic bias, the authors introduce a reweighting strategy that ensures equitable performance across age, gender, and ethnic groups.

 Data preprocessing involved fuzzy imputation, normalization, and categorical encoding, while hyperparameters were tuned through grid search. Evaluations using 10-fold cross-validation achieved ~98.2% accuracy, 98.1% F1-score, and 99.1% AUC, surpassing traditional machine learning and recent deep learning baselines. SHAP results revealed chest pain type, ST depression, oldpeak, thalassemia, and maximum heart rate as dominant predictors. The findings demonstrate that X-TLRABiLSTM is a reliable and transparent tool for clinical decision support in cardiovascular disease prognosis. The paper fulfills all the required criteria, including technical soundness and content quality. It is also up to the mark and can be published here.

Is the work clearly and accurately presented and does it cite the current literature?

Yes

If applicable, is the statistical analysis and its interpretation appropriate?

Yes

Are all the source data underlying the results available to ensure full reproducibility?

No source data required

Is the study design appropriate and is the work technically sound?

Yes

Are the conclusions drawn adequately supported by the results?

Yes

Are sufficient details of methods and analysis provided to allow replication by others?

Yes

Reviewer Expertise:

Machine Learning, Deep Learning, Computer Vision, AI

We confirm that we have read this submission and believe that we have an appropriate level of expertise to confirm that it is of an acceptable scientific standard.

# Response

**Published:** 2025-09-16

**Authors:** VIJAYA ARJUNAN RANGANATHAN

**Affiliations:** School of Computer Engineering, Manipal Academy of Higher Education, Manipal, Karnataka, India

We sincerely thank the reviewer for taking the time to evaluate our manuscript and approving the work. We are grateful for the approval and encouragement, which supports the value of our work.
